# 2-(3-Cyano-4-methyl-5,5-diphenyl-5*H*-furan-2-yl­idene)malono­nitrile

**DOI:** 10.1107/S1600536813020849

**Published:** 2013-08-03

**Authors:** M. Delower H. Bhuiyan, Graeme J. Gainsford, Andrew Kay, Jack Anderson

**Affiliations:** a Callaghan Innovation Research Limited, PO Box 31-310, Lower Hutt, New Zealand; b Industrial Research Limited, PO Box 31-310, Lower Hutt, New Zealand

## Abstract

The title compound, C_21_H_13_N_3_O, crystallizes with two independent molecules with similar conformations per asymmetric unit. The dihydrofuran rings are essentially planar with maximum deviations of 0.017 (1) and 0.006 (1) Å for the O atoms. The dihedral angles between the di­hydro­furan ring and the attached phenyl rings are 79.90 (6) and 82.07 (6)° in one mol­ecule and 79.36 (6) and 72.26 (6)° in the other. In the crystal, the molecules are linked by weak C—H⋯π and C—H⋯N inter­actions similar to those in other closely related crystals. The replacement of appended methyl by phenyl groups has not significantly affected the dihydrofuran ring structure or the crystal packing interactions.

## Related literature

For general background to NLO chromophores, see: Smith *et al.* (2006 ▶, 2010 ▶); Carey *et al.* (2002 ▶); Kay *et al.* (2004 ▶). For details of the synthesis, see: Anderson (2009 ▶). For related structures, see: Anderson (2009 ▶); Gainsford *et al.* (2011 ▶); Li *et al.* (2005 ▶); Liao *et al.* (2005 ▶). For geometric analysis of structures, see: Spek (2009 ▶). For a description of the Cambridge Structural Database, see: Allen (2002 ▶).
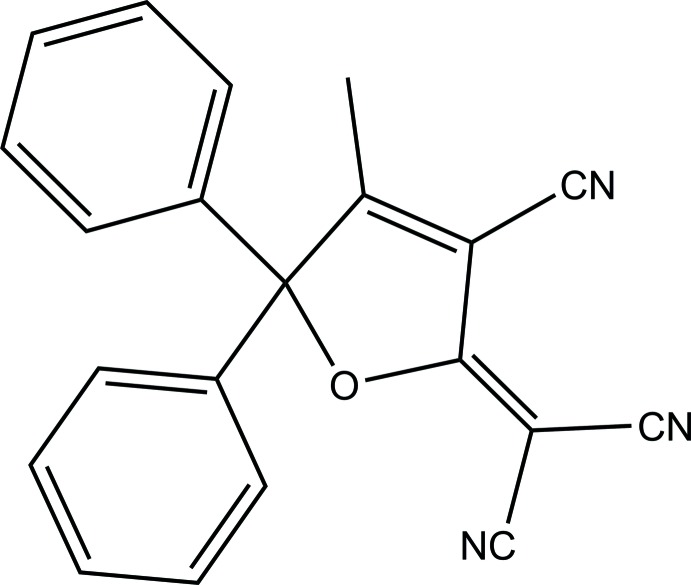


## Experimental

### 

#### Crystal data

C_21_H_13_N_3_O
*M* *_r_* = 323.34
Triclinic, 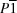

*a* = 9.2308 (3) Å
*b* = 12.5991 (4) Å
*c* = 14.3043 (4) Å
α = 89.954 (2)°
β = 89.052 (2)°
γ = 79.233 (2)°
*V* = 1634.07 (9) Å^3^
*Z* = 4
Mo *K*α radiation
μ = 0.08 mm^−1^
*T* = 123 K
0.36 × 0.32 × 0.29 mm

#### Data collection

Bruker APEXII CCD diffractometer
Absorption correction: multi-scan (*SADABS*; Bruker, 2005 ▶) *T*_min_ = 0.651, *T*_max_ = 0.746
45305 measured reflections
9930 independent reflections
7329 reflections with *I* > 2σ(*I*)
*R*_int_ = 0.042

#### Refinement

*R*[*F* ^2^ > 2σ(*F* ^2^)] = 0.045
*wR*(*F* ^2^) = 0.119
*S* = 1.04
9930 reflections
453 parameters
H-atom parameters constrained
Δρ_max_ = 0.35 e Å^−3^
Δρ_min_ = −0.24 e Å^−3^

### 

Data collection: *APEX2* (Bruker, 2005 ▶); cell refinement: *SAINT* (Bruker, 2005 ▶); data reduction: *SAINT*; program(s) used to solve structure: *SHELXS97* (Sheldrick, 2008 ▶); program(s) used to refine structure: *SHELXL97* (Sheldrick, 2008 ▶); molecular graphics: *ORTEP-3 for Windows* (Farrugia, 2012 ▶) and *Mercury* (Macrae *et al.*, 2006 ▶); software used to prepare material for publication: *SHELXL97* and *PLATON* (Spek, 2009 ▶).

## Supplementary Material

Crystal structure: contains datablock(s) global, I. DOI: 10.1107/S1600536813020849/rz5081sup1.cif

Structure factors: contains datablock(s) I. DOI: 10.1107/S1600536813020849/rz5081Isup2.hkl

Supplementary material file. DOI: 10.1107/S1600536813020849/rz5081Isup3.cml

Additional supplementary materials:  crystallographic information; 3D view; checkCIF report

## Figures and Tables

**Table 1:** Hydrogen-bond geometry (Å, °) *Cg*1 is the centroid of the C10–C15 phenyl ring

*D*—H⋯*A*	*D*—H	H⋯*A*	*D*⋯*A*	*D*—H⋯*A*
C20′—H20′⋯*Cg*1^i^	0.95	2.69	3.4041 (14)	133
C9—H9*B*⋯N3′	0.98	2.70	3.4560 (14)	134

Symmetry code: (i) 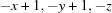.

**Fig. 1.**
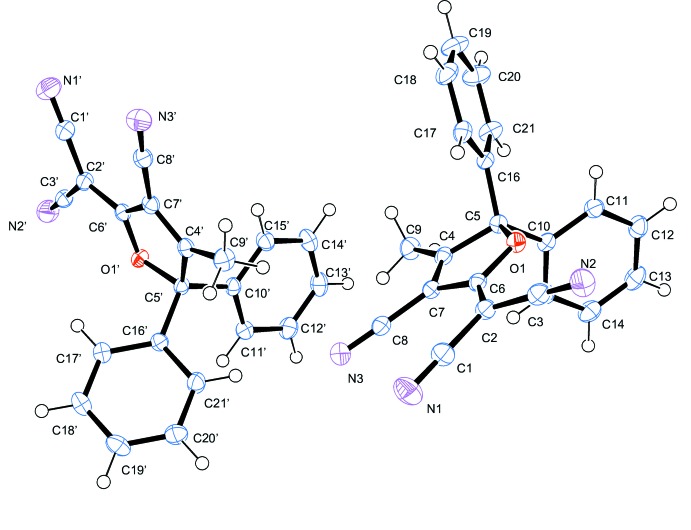
Content of the asymmetric unit of the title compound with displacement ellipsoids drawn at the 50% probability level. The partially obscured atom C15 is not labelled.

**Fig. 2.**
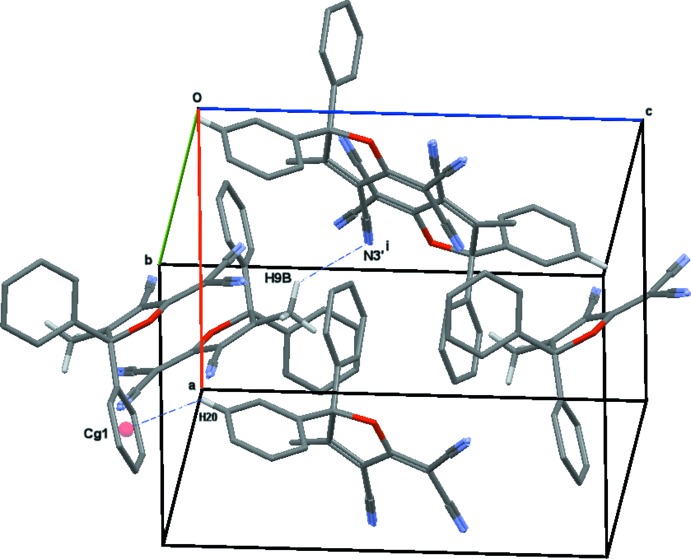
Packing diagram of the title compound with one C–H···π and C(methyl)–H···N(cyano) interaction shown as dashed blue lines. H atoms not involved in intermolecular contacts are excluded except on methyl C9. Cg1 is the centroid of the phenyl ring (C10–C15) at symmetry 1-x, 1-y, -z. Symmetry code: (i) -1+x, y, z.

## supplementary crystallographic information

### 1. Comment

Organic non-linear optical (NLO) chromophores consist of donor and acceptor units connecting through π-conjugation. To realise a strong NLO response, chromophores need to be in uniform alignment. Unfortunately organic chromophores have a tendency to aggregate rather than align due to their highly polar nature (Smith *et al.*, 2006). These push-pull chromophores can also exist as two rotomeric (*cis* and *trans*) forms (Kay *et al.*, 2004). As a result, these chromophores are difficult to crystallize, therefore they may not be able to be used in devices that require crystals such as terahertz wave emitters (Carey *et al.*, 2002). Consequently it is of interest to design and synthesize molecules which will be much less prone to aggregation and isomerization. It has been found that through changing the shape of the chromophore molecules, aggregation can be minimized. The most successful strategy has been to add bulky pendant groups onto the donor end (Smith *et al.*, 2010). We have reported our previous attempts with two benzyloxyphenyl groups (Gainsford *et al.*, 2011; Anderson, 2009) and report here the structure of a related acceptor unit with two phenyl groups.

The asymmetric unit of the title compound (I) contains two independent, nearly identical, 2-(3-cyano-4-methyl-5,5-diphenyl-5*H*-furan-2-ylidene)malononitrile molecules (Fig. 1). The second molecule (1') has identical labels with an appended prime (*e.g.* C10 and C10'); the r.m.s. bond and angle fits are 0.003 Å and 0.48° (Spek, 2009). The five-membered dihydrofuran rings (atoms C4-C7/O1, hereafter plane 1) are planar (maximum deviation 0.017 (1) Å for O1) with the appended cyano groups almost coplanar (maximum deviation 0.093 (1) Å for N1). The 5,5-dimethyl adduct (Li *et al.*, 2005; CSD refcode PANLUM), in which plane 1 was constrained to a crystallographic mirror plane, has identical ring dimensions with the exception of the C4–C5 bond which is just significantly longer here (by 0.013 (4) Å). This marginally longer distance seems the exception and is not observed in related molecules [Allen (2002); CSD version 5.34 with May 2013 updates: *e.g.* KAJCII and KATCEE, 1.518 and 1.536, 1.537 Å respectively (Liao *et al.*, 2005); YAHKUP 1.512 Å (Gainsford *et al.*, 2011)].

The only significant conformational differences between the two molecules concern the interplanar angles between the phenyl rings and plane 1, corresponding to the different torsion angles (C4–C5–C10–C15 = -21.21 (15)°; O1–C5–C16–C17 = -30.62 (13)°; C4'–C5'–C10'–C15' = -30.22 (14)°; O1'–C5'–C16'–C17' = -21.91 (15)°). The plane 1 angle to phenyl plane (C10–C15) is 79.90 (6) and 82.07 (6)° for molecules 1 and 1' while the other phenyl plane (C16–C21) makes interplane angle of 79.36 (6) and 72.26 (6)° for molecules 1 and 1'.

Lattice binding is provided mainly by one non-classical C–H···π interaction (Table 1, Figure 2 where *Cg*1 is the centroid of the C10–C15 ring), the dominant binding interaction type in related compound YAHKUP (Gainsford *et al.*, 2011). As in PANLUM, a weak methyl*C*–*H*···*N*(cyano) interaction is observed. Other *C*–*H*···*N*(cyano), C–H···π and cyano···cyano very weak interactions even closer to van der Waal's contact distances are also present. Overall, the effect of the phenyl for methyl replacement on C5 has given insignificant molecular structural and cystal packing alignment affects.

### 2. Experimental

The title compound was prepared by the condensation of 1-hydroxy-1,1-diphenylpropan-2-one with 4 equivalents of malononitrile over 10 days as described in Anderson (2009). A small portion was recrystallized in dichloromethane and acetone (1:1) mixture to give colourless crystals. M.p. 223 °C. Found: C, 77.70; H, 3.91; N, 13.06, C_21_H_13_N_3_O requires C, 78.00; H, 4.05; N, 13.00%. Found: *M*^+^ m/*z* 323.1059 C_21_H_13_N_3_O requires: *M*+ m/*z* 323.1056 (Δ 0.9 p.p.m.). ^1^H NMR– (300 MHz, CDCl_3_) δ (p.p.m.): 2.41 (s, 3H), 7.18 (d, *J* 9.6 Hz, 4H), 7.51–7.47 (m, 6H). ^13^C NMR– (75 MHz, CDCl_3_) δ (p.p.m.): 16.2 (CH_3_), 59.5 (C_Q_), 105.6 (C_Q_), 106.4 (C_Q_), 109.0 (C_Q_), 110.2 (C_Q_), 110.7 (C_Q_), 127.3 (CH), 129.5 (CH), 130.6 (CH), 134.9 (C_Q_), 175.1 (C_Q_), 180.2 (C_Q_).

### 3. Refinement

All carbon-bound H atoms were constrained to their expected geometries [C—H 0.95, 0.98 Å] and refined with *U*_iso_ 1.2 times the *U*_eq_ of their parent atom. All other non-hydrogen atoms were refined with anisotropic thermal parameters.

### Crystal data

**Table:**

C_21_H_13_N_3_O	*Z* = 4
*M**_r_* = 323.34	*F*(000) = 672
Triclinic, *P*1	*D*_x_ = 1.314 Mg m^−^^3^
Hall symbol: -P 1	Mo *K*α radiation, λ = 0.71073 Å
*a* = 9.2308 (3) Å	Cell parameters from 9027 reflections
*b* = 12.5991 (4) Å	θ = 2.2–30.5°
*c* = 14.3043 (4) Å	µ = 0.08 mm^−^^1^
α = 89.954 (2)°	*T* = 123 K
β = 89.052 (2)°	Block, colourless
γ = 79.233 (2)°	0.36 × 0.32 × 0.29 mm
*V* = 1634.07 (9) Å^3^	

### Data collection

**Table:**

Bruker APEXII CCD diffractometer	9930 independent reflections
Radiation source: fine-focus sealed tube	7329 reflections with *I* > 2σ(*I*)
Graphite monochromator	*R*_int_ = 0.042
Detector resolution: 8.333 pixels mm^-1^	θ_max_ = 30.7°, θ_min_ = 1.4°
φ and ω scans	*h* = −13→13
Absorption correction: multi-scan (*SADABS*; Bruker, 2005)	*k* = −18→18
*T*_min_ = 0.651, *T*_max_ = 0.746	*l* = −20→20
45305 measured reflections	

### Refinement

**Table:**

Refinement on *F*^2^	Primary atom site location: structure-invariant direct methods
Least-squares matrix: full	Secondary atom site location: difference Fourier map
*R*[*F*^2^ > 2σ(*F*^2^)] = 0.045	Hydrogen site location: inferred from neighbouring sites
*wR*(*F*^2^) = 0.119	H-atom parameters constrained
*S* = 1.04	*w* = 1/[σ^2^(*F*_o_^2^) + (0.0512*P*)^2^ + 0.3811*P*] where *P* = (*F*_o_^2^ + 2*F*_c_^2^)/3
9930 reflections	(Δ/σ)_max_ < 0.001
453 parameters	Δρ_max_ = 0.35 e Å^−^^3^
0 restraints	Δρ_min_ = −0.24 e Å^−^^3^

### Special details

**Table:**

Geometry. All e.s.d.'s (except the e.s.d. in the dihedral angle between two l.s. planes) are estimated using the full covariance matrix. The cell e.s.d.'s are taken into account individually in the estimation of e.s.d.'s in distances, angles and torsion angles; correlations between e.s.d.'s in cell parameters are only used when they are defined by crystal symmetry. An approximate (isotropic) treatment of cell e.s.d.'s is used for estimating e.s.d.'s involving l.s. planes.
Refinement. Refinement of *F*^2^ against ALL reflections. The weighted *R*-factor *wR* and goodness of fit *S* are based on *F*^2^, conventional *R*-factors *R* are based on *F*, with *F* set to zero for negative *F*^2^. The threshold expression of *F*^2^ > σ(*F*^2^) is used only for calculating *R*-factors(gt) *etc*. and is not relevant to the choice of reflections for refinement. *R*-factors based on *F*^2^ are statistically about twice as large as those based on *F*, and *R*- factors based on ALL data will be even larger.

### Fractional atomic coordinates and isotropic or equivalent isotropic displacement parameters (Å^2^)

**Table:**

	*x*	*y*	*z*	*U*_iso_*/*U*_eq_	
O1	0.34806 (9)	0.80434 (6)	0.09747 (5)	0.01913 (17)	
N1	0.71127 (14)	0.64480 (10)	−0.12562 (9)	0.0387 (3)	
N2	0.41086 (13)	0.95957 (10)	−0.09834 (8)	0.0315 (3)	
N3	0.68541 (13)	0.46791 (10)	0.06030 (8)	0.0320 (3)	
C1	0.62087 (14)	0.70061 (10)	−0.08395 (9)	0.0255 (3)	
C2	0.50822 (13)	0.77028 (10)	−0.03197 (8)	0.0204 (2)	
C3	0.45366 (13)	0.87575 (10)	−0.06807 (8)	0.0228 (2)	
C4	0.40415 (12)	0.64052 (9)	0.17749 (8)	0.0194 (2)	
C5	0.30203 (12)	0.74917 (9)	0.18178 (8)	0.0176 (2)	
C6	0.45279 (12)	0.73785 (9)	0.04999 (8)	0.0183 (2)	
C7	0.49044 (12)	0.63614 (9)	0.10052 (8)	0.0193 (2)	
C8	0.60060 (13)	0.54476 (10)	0.07557 (8)	0.0225 (2)	
C9	0.40783 (15)	0.55820 (10)	0.25144 (9)	0.0269 (3)	
H9A	0.4637	0.4889	0.2288	0.032*	
H9B	0.3069	0.5502	0.2677	0.032*	
H9C	0.4553	0.5808	0.3069	0.032*	
C10	0.14038 (12)	0.74436 (9)	0.16821 (8)	0.0188 (2)	
C11	0.03222 (13)	0.83316 (10)	0.19229 (8)	0.0225 (2)	
H11	0.0589	0.8940	0.2219	0.027*	
C12	−0.11448 (14)	0.83266 (11)	0.17303 (9)	0.0278 (3)	
H12	−0.1884	0.8930	0.1899	0.033*	
C13	−0.15301 (14)	0.74430 (12)	0.12926 (9)	0.0301 (3)	
H13	−0.2535	0.7441	0.1162	0.036*	
C14	−0.04624 (15)	0.65645 (11)	0.10437 (9)	0.0288 (3)	
H14	−0.0733	0.5963	0.0738	0.035*	
C15	0.10062 (14)	0.65600 (10)	0.12400 (9)	0.0238 (2)	
H15	0.1740	0.5953	0.1072	0.029*	
C16	0.33659 (12)	0.80840 (9)	0.26874 (8)	0.0186 (2)	
C17	0.45540 (13)	0.86160 (10)	0.26897 (9)	0.0226 (2)	
H17	0.5119	0.8654	0.2134	0.027*	
C18	0.49144 (15)	0.90922 (11)	0.35082 (9)	0.0287 (3)	
H18	0.5728	0.9456	0.3512	0.034*	
C19	0.40914 (17)	0.90379 (12)	0.43177 (9)	0.0337 (3)	
H19	0.4334	0.9371	0.4875	0.040*	
C20	0.29189 (17)	0.85014 (12)	0.43188 (9)	0.0329 (3)	
H20	0.2352	0.8468	0.4875	0.040*	
C21	0.25681 (15)	0.80120 (10)	0.35100 (8)	0.0255 (3)	
H21	0.1778	0.7625	0.3517	0.031*	
O1'	0.98981 (9)	0.20107 (6)	0.40488 (5)	0.01929 (17)	
N1'	1.26942 (15)	0.36533 (11)	0.61930 (9)	0.0397 (3)	
N2'	1.13037 (14)	0.05064 (10)	0.60095 (8)	0.0346 (3)	
N3'	1.16038 (13)	0.53580 (9)	0.43102 (8)	0.0302 (2)	
C1'	1.20774 (14)	0.30913 (11)	0.57926 (9)	0.0265 (3)	
C2'	1.13161 (13)	0.23860 (10)	0.53017 (8)	0.0214 (2)	
C3'	1.12990 (14)	0.13375 (10)	0.56895 (8)	0.0236 (2)	
C4'	0.97057 (12)	0.35981 (9)	0.31831 (8)	0.0192 (2)	
C5'	0.92300 (12)	0.25132 (9)	0.31831 (8)	0.0181 (2)	
C6'	1.06105 (12)	0.26850 (9)	0.44854 (8)	0.0183 (2)	
C7'	1.05045 (12)	0.36764 (9)	0.39479 (8)	0.0194 (2)	
C8'	1.11373 (13)	0.45944 (10)	0.41679 (8)	0.0220 (2)	
C9'	0.93503 (15)	0.43960 (10)	0.24239 (9)	0.0262 (3)	
H9'A	0.9634	0.5077	0.2607	0.031*	
H9'B	0.8289	0.4520	0.2309	0.031*	
H9'C	0.9893	0.4120	0.1853	0.031*	
C10'	0.75678 (12)	0.26066 (9)	0.33075 (8)	0.0187 (2)	
C11'	0.68589 (13)	0.18118 (10)	0.29681 (9)	0.0231 (2)	
H11'	0.7401	0.1214	0.2630	0.028*	
C12'	0.53511 (14)	0.18933 (11)	0.31243 (9)	0.0276 (3)	
H12'	0.4859	0.1359	0.2879	0.033*	
C13'	0.45660 (14)	0.27472 (11)	0.36351 (10)	0.0290 (3)	
H13'	0.3537	0.2798	0.3741	0.035*	
C14'	0.52747 (14)	0.35269 (11)	0.39920 (10)	0.0291 (3)	
H14'	0.4737	0.4106	0.4353	0.035*	
C15'	0.67724 (14)	0.34638 (10)	0.38225 (9)	0.0241 (2)	
H15'	0.7256	0.4007	0.4059	0.029*	
C16'	0.99343 (13)	0.18644 (9)	0.23453 (8)	0.0189 (2)	
C17'	1.13164 (13)	0.12020 (9)	0.24063 (9)	0.0217 (2)	
H17'	1.1791	0.1096	0.2992	0.026*	
C18'	1.19993 (14)	0.06974 (10)	0.16117 (9)	0.0262 (3)	
H18'	1.2949	0.0254	0.1654	0.031*	
C19'	1.13090 (16)	0.08335 (10)	0.07570 (9)	0.0288 (3)	
H19'	1.1776	0.0478	0.0216	0.035*	
C20'	0.99357 (15)	0.14899 (11)	0.06954 (9)	0.0287 (3)	
H20'	0.9458	0.1584	0.0110	0.034*	
C21'	0.92545 (14)	0.20087 (10)	0.14786 (8)	0.0252 (3)	
H21'	0.8317	0.2467	0.1428	0.030*	

### Atomic displacement parameters (Å^2^)

**Table:**

	*U*^11^	*U*^22^	*U*^33^	*U*^12^	*U*^13^	*U*^23^
O1	0.0182 (4)	0.0211 (4)	0.0176 (4)	−0.0025 (3)	0.0024 (3)	0.0021 (3)
N1	0.0364 (7)	0.0400 (7)	0.0369 (7)	−0.0012 (6)	0.0125 (5)	−0.0044 (6)
N2	0.0309 (6)	0.0339 (6)	0.0282 (6)	−0.0025 (5)	0.0041 (5)	0.0049 (5)
N3	0.0288 (6)	0.0323 (6)	0.0317 (6)	0.0029 (5)	−0.0057 (5)	−0.0042 (5)
C1	0.0244 (6)	0.0287 (6)	0.0235 (6)	−0.0057 (5)	0.0027 (5)	0.0005 (5)
C2	0.0182 (5)	0.0246 (6)	0.0184 (5)	−0.0042 (4)	0.0005 (4)	−0.0005 (4)
C3	0.0206 (6)	0.0305 (6)	0.0177 (5)	−0.0060 (5)	0.0025 (4)	−0.0003 (5)
C4	0.0180 (5)	0.0202 (5)	0.0208 (5)	−0.0057 (4)	−0.0035 (4)	0.0002 (4)
C5	0.0172 (5)	0.0196 (5)	0.0160 (5)	−0.0039 (4)	0.0017 (4)	0.0020 (4)
C6	0.0153 (5)	0.0216 (5)	0.0184 (5)	−0.0045 (4)	−0.0021 (4)	−0.0018 (4)
C7	0.0171 (5)	0.0206 (5)	0.0204 (5)	−0.0039 (4)	−0.0027 (4)	−0.0016 (4)
C8	0.0193 (5)	0.0258 (6)	0.0225 (6)	−0.0045 (5)	−0.0035 (4)	−0.0022 (5)
C9	0.0306 (7)	0.0238 (6)	0.0258 (6)	−0.0040 (5)	−0.0005 (5)	0.0060 (5)
C10	0.0170 (5)	0.0241 (5)	0.0161 (5)	−0.0055 (4)	−0.0003 (4)	0.0021 (4)
C11	0.0203 (5)	0.0264 (6)	0.0211 (5)	−0.0049 (5)	0.0002 (4)	−0.0015 (5)
C12	0.0188 (6)	0.0365 (7)	0.0265 (6)	−0.0013 (5)	0.0000 (5)	0.0007 (5)
C13	0.0187 (6)	0.0440 (8)	0.0297 (6)	−0.0110 (5)	−0.0043 (5)	0.0040 (6)
C14	0.0281 (6)	0.0318 (7)	0.0301 (6)	−0.0146 (5)	−0.0055 (5)	0.0011 (5)
C15	0.0234 (6)	0.0238 (6)	0.0254 (6)	−0.0070 (5)	−0.0015 (5)	0.0000 (5)
C16	0.0180 (5)	0.0197 (5)	0.0180 (5)	−0.0032 (4)	−0.0026 (4)	0.0010 (4)
C17	0.0188 (5)	0.0234 (6)	0.0259 (6)	−0.0047 (4)	−0.0024 (5)	0.0033 (5)
C18	0.0287 (6)	0.0274 (6)	0.0330 (7)	−0.0121 (5)	−0.0109 (5)	0.0032 (5)
C19	0.0466 (8)	0.0355 (7)	0.0231 (6)	−0.0175 (6)	−0.0107 (6)	−0.0007 (5)
C20	0.0451 (8)	0.0385 (7)	0.0185 (6)	−0.0164 (6)	0.0006 (6)	0.0005 (5)
C21	0.0287 (6)	0.0306 (6)	0.0204 (5)	−0.0135 (5)	−0.0011 (5)	0.0015 (5)
O1'	0.0198 (4)	0.0208 (4)	0.0176 (4)	−0.0045 (3)	−0.0035 (3)	0.0023 (3)
N1'	0.0412 (7)	0.0389 (7)	0.0397 (7)	−0.0079 (6)	−0.0131 (6)	−0.0056 (6)
N2'	0.0397 (7)	0.0325 (6)	0.0303 (6)	−0.0033 (5)	−0.0062 (5)	0.0042 (5)
N3'	0.0302 (6)	0.0310 (6)	0.0312 (6)	−0.0109 (5)	0.0057 (5)	−0.0038 (5)
C1'	0.0250 (6)	0.0289 (6)	0.0243 (6)	−0.0013 (5)	−0.0044 (5)	−0.0013 (5)
C2'	0.0192 (5)	0.0246 (6)	0.0195 (5)	−0.0020 (4)	−0.0017 (4)	−0.0006 (4)
C3'	0.0229 (6)	0.0276 (6)	0.0184 (5)	−0.0002 (5)	−0.0034 (5)	−0.0010 (5)
C4'	0.0154 (5)	0.0209 (5)	0.0203 (5)	−0.0016 (4)	0.0033 (4)	0.0007 (4)
C5'	0.0175 (5)	0.0201 (5)	0.0158 (5)	−0.0011 (4)	−0.0022 (4)	0.0023 (4)
C6'	0.0141 (5)	0.0214 (5)	0.0188 (5)	−0.0019 (4)	0.0020 (4)	−0.0018 (4)
C7'	0.0161 (5)	0.0213 (5)	0.0208 (5)	−0.0036 (4)	0.0030 (4)	−0.0005 (4)
C8'	0.0188 (5)	0.0251 (6)	0.0219 (6)	−0.0042 (5)	0.0032 (4)	−0.0009 (5)
C9'	0.0293 (6)	0.0250 (6)	0.0241 (6)	−0.0046 (5)	−0.0004 (5)	0.0061 (5)
C10'	0.0167 (5)	0.0215 (5)	0.0172 (5)	−0.0015 (4)	−0.0001 (4)	0.0031 (4)
C11'	0.0214 (6)	0.0240 (6)	0.0234 (6)	−0.0030 (5)	0.0003 (5)	−0.0010 (5)
C12'	0.0221 (6)	0.0299 (6)	0.0325 (7)	−0.0087 (5)	−0.0021 (5)	0.0027 (5)
C13'	0.0177 (6)	0.0339 (7)	0.0344 (7)	−0.0025 (5)	0.0028 (5)	0.0059 (6)
C14'	0.0225 (6)	0.0284 (6)	0.0335 (7)	0.0019 (5)	0.0060 (5)	0.0001 (5)
C15'	0.0215 (6)	0.0234 (6)	0.0268 (6)	−0.0029 (5)	0.0013 (5)	−0.0013 (5)
C16'	0.0185 (5)	0.0196 (5)	0.0187 (5)	−0.0037 (4)	0.0012 (4)	0.0004 (4)
C17'	0.0196 (5)	0.0208 (5)	0.0244 (6)	−0.0029 (4)	0.0004 (4)	0.0020 (5)
C18'	0.0235 (6)	0.0215 (6)	0.0313 (6)	0.0008 (5)	0.0060 (5)	0.0021 (5)
C19'	0.0347 (7)	0.0257 (6)	0.0245 (6)	−0.0023 (5)	0.0103 (5)	−0.0019 (5)
C20'	0.0331 (7)	0.0337 (7)	0.0183 (5)	−0.0035 (6)	0.0014 (5)	0.0000 (5)
C21'	0.0220 (6)	0.0308 (6)	0.0208 (6)	0.0002 (5)	−0.0012 (5)	0.0022 (5)

### Geometric parameters (Å, º)

**Table:**

O1—C6	1.3320 (14)	O1'—C6'	1.3295 (14)
O1—C5	1.4866 (13)	O1'—C5'	1.4808 (12)
N1—C1	1.1450 (17)	N1'—C1'	1.1469 (18)
N2—C3	1.1437 (16)	N2'—C3'	1.1417 (17)
N3—C8	1.1443 (16)	N3'—C8'	1.1448 (16)
C1—C2	1.4280 (18)	C1'—C2'	1.4239 (18)
C2—C6	1.3642 (16)	C2'—C6'	1.3647 (15)
C2—C3	1.4295 (16)	C2'—C3'	1.4351 (17)
C4—C7	1.3432 (17)	C4'—C7'	1.3432 (16)
C4—C9	1.4772 (16)	C4'—C9'	1.4779 (15)
C4—C5	1.5107 (16)	C4'—C5'	1.5118 (16)
C5—C16	1.5197 (16)	C5'—C16'	1.5180 (16)
C5—C10	1.5202 (16)	C5'—C10'	1.5243 (15)
C6—C7	1.4580 (15)	C6'—C7'	1.4547 (15)
C7—C8	1.4277 (17)	C7'—C8'	1.4273 (17)
C9—H9A	0.9800	C9'—H9'A	0.9800
C9—H9B	0.9800	C9'—H9'B	0.9800
C9—H9C	0.9800	C9'—H9'C	0.9800
C10—C15	1.3912 (17)	C10'—C11'	1.3866 (17)
C10—C11	1.3926 (17)	C10'—C15'	1.3911 (17)
C11—C12	1.3875 (17)	C11'—C12'	1.3907 (17)
C11—H11	0.9500	C11'—H11'	0.9500
C12—C13	1.384 (2)	C12'—C13'	1.3816 (19)
C12—H12	0.9500	C12'—H12'	0.9500
C13—C14	1.380 (2)	C13'—C14'	1.380 (2)
C13—H13	0.9500	C13'—H13'	0.9500
C14—C15	1.3879 (17)	C14'—C15'	1.3872 (18)
C14—H14	0.9500	C14'—H14'	0.9500
C15—H15	0.9500	C15'—H15'	0.9500
C16—C17	1.3881 (16)	C16'—C17'	1.3919 (16)
C16—C21	1.3898 (17)	C16'—C21'	1.3952 (16)
C17—C18	1.3894 (18)	C17'—C18'	1.3861 (18)
C17—H17	0.9500	C17'—H17'	0.9500
C18—C19	1.383 (2)	C18'—C19'	1.3837 (19)
C18—H18	0.9500	C18'—H18'	0.9500
C19—C20	1.379 (2)	C19'—C20'	1.3822 (19)
C19—H19	0.9500	C19'—H19'	0.9500
C20—C21	1.3826 (18)	C20'—C21'	1.3792 (18)
C20—H20	0.9500	C20'—H20'	0.9500
C21—H21	0.9500	C21'—H21'	0.9500
			
C6—O1—C5	110.17 (8)	C6'—O1'—C5'	110.25 (8)
N1—C1—C2	179.94 (19)	N1'—C1'—C2'	179.47 (14)
C6—C2—C1	121.39 (11)	C6'—C2'—C1'	121.78 (11)
C6—C2—C3	120.28 (11)	C6'—C2'—C3'	120.02 (11)
C1—C2—C3	118.33 (11)	C1'—C2'—C3'	118.20 (10)
N2—C3—C2	178.90 (14)	N2'—C3'—C2'	178.76 (14)
C7—C4—C9	127.97 (11)	C7'—C4'—C9'	127.80 (11)
C7—C4—C5	109.10 (10)	C7'—C4'—C5'	109.09 (10)
C9—C4—C5	122.86 (11)	C9'—C4'—C5'	123.09 (10)
O1—C5—C4	102.37 (9)	O1'—C5'—C4'	102.35 (9)
O1—C5—C16	109.19 (9)	O1'—C5'—C16'	109.11 (8)
C4—C5—C16	108.53 (9)	C4'—C5'—C16'	108.89 (9)
O1—C5—C10	105.72 (8)	O1'—C5'—C10'	106.34 (8)
C4—C5—C10	114.22 (10)	C4'—C5'—C10'	112.82 (9)
C16—C5—C10	115.82 (10)	C16'—C5'—C10'	116.33 (10)
O1—C6—C2	120.36 (10)	O1'—C6'—C2'	120.27 (10)
O1—C6—C7	109.26 (10)	O1'—C6'—C7'	109.49 (9)
C2—C6—C7	130.38 (11)	C2'—C6'—C7'	130.22 (11)
C4—C7—C8	123.65 (11)	C4'—C7'—C8'	124.09 (11)
C4—C7—C6	109.02 (10)	C4'—C7'—C6'	108.81 (10)
C8—C7—C6	127.34 (11)	C8'—C7'—C6'	127.10 (10)
N3—C8—C7	175.48 (14)	N3'—C8'—C7'	176.62 (13)
C4—C9—H9A	109.5	C4'—C9'—H9'A	109.5
C4—C9—H9B	109.5	C4'—C9'—H9'B	109.5
H9A—C9—H9B	109.5	H9'A—C9'—H9'B	109.5
C4—C9—H9C	109.5	C4'—C9'—H9'C	109.5
H9A—C9—H9C	109.5	H9'A—C9'—H9'C	109.5
H9B—C9—H9C	109.5	H9'B—C9'—H9'C	109.5
C15—C10—C11	119.65 (11)	C11'—C10'—C15'	119.72 (11)
C15—C10—C5	120.37 (11)	C11'—C10'—C5'	120.81 (10)
C11—C10—C5	119.70 (11)	C15'—C10'—C5'	119.32 (11)
C12—C11—C10	119.95 (12)	C10'—C11'—C12'	119.71 (12)
C12—C11—H11	120.0	C10'—C11'—H11'	120.1
C10—C11—H11	120.0	C12'—C11'—H11'	120.1
C13—C12—C11	119.97 (12)	C13'—C12'—C11'	120.31 (12)
C13—C12—H12	120.0	C13'—C12'—H12'	119.8
C11—C12—H12	120.0	C11'—C12'—H12'	119.8
C14—C13—C12	120.39 (12)	C14'—C13'—C12'	120.11 (12)
C14—C13—H13	119.8	C14'—C13'—H13'	119.9
C12—C13—H13	119.8	C12'—C13'—H13'	119.9
C13—C14—C15	119.99 (12)	C13'—C14'—C15'	119.93 (12)
C13—C14—H14	120.0	C13'—C14'—H14'	120.0
C15—C14—H14	120.0	C15'—C14'—H14'	120.0
C14—C15—C10	120.03 (12)	C14'—C15'—C10'	120.18 (12)
C14—C15—H15	120.0	C14'—C15'—H15'	119.9
C10—C15—H15	120.0	C10'—C15'—H15'	119.9
C17—C16—C21	119.62 (11)	C17'—C16'—C21'	119.14 (11)
C17—C16—C5	120.49 (11)	C17'—C16'—C5'	120.97 (10)
C21—C16—C5	119.64 (10)	C21'—C16'—C5'	119.62 (10)
C16—C17—C18	119.77 (12)	C18'—C17'—C16'	119.95 (11)
C16—C17—H17	120.1	C18'—C17'—H17'	120.0
C18—C17—H17	120.1	C16'—C17'—H17'	120.0
C19—C18—C17	120.14 (12)	C19'—C18'—C17'	120.54 (11)
C19—C18—H18	119.9	C19'—C18'—H18'	119.7
C17—C18—H18	119.9	C17'—C18'—H18'	119.7
C20—C19—C18	120.17 (13)	C20'—C19'—C18'	119.58 (12)
C20—C19—H19	119.9	C20'—C19'—H19'	120.2
C18—C19—H19	119.9	C18'—C19'—H19'	120.2
C19—C20—C21	119.97 (13)	C21'—C20'—C19'	120.41 (12)
C19—C20—H20	120.0	C21'—C20'—H20'	119.8
C21—C20—H20	120.0	C19'—C20'—H20'	119.8
C20—C21—C16	120.30 (12)	C20'—C21'—C16'	120.36 (11)
C20—C21—H21	119.9	C20'—C21'—H21'	119.8
C16—C21—H21	119.9	C16'—C21'—H21'	119.8
			
C6—O1—C5—C4	2.74 (11)	C6'—O1'—C5'—C4'	1.01 (11)
C6—O1—C5—C16	117.63 (10)	C6'—O1'—C5'—C16'	116.26 (10)
C6—O1—C5—C10	−117.13 (10)	C6'—O1'—C5'—C10'	−117.54 (10)
C7—C4—C5—O1	−1.50 (12)	C7'—C4'—C5'—O1'	−0.64 (12)
C9—C4—C5—O1	175.57 (10)	C9'—C4'—C5'—O1'	177.60 (10)
C7—C4—C5—C16	−116.87 (11)	C7'—C4'—C5'—C16'	−116.05 (10)
C9—C4—C5—C16	60.21 (14)	C9'—C4'—C5'—C16'	62.19 (13)
C7—C4—C5—C10	112.25 (11)	C7'—C4'—C5'—C10'	113.21 (11)
C9—C4—C5—C10	−70.67 (14)	C9'—C4'—C5'—C10'	−68.54 (14)
C5—O1—C6—C2	177.73 (10)	C5'—O1'—C6'—C2'	−179.83 (10)
C5—O1—C6—C7	−2.95 (12)	C5'—O1'—C6'—C7'	−1.00 (12)
C1—C2—C6—O1	179.78 (11)	C1'—C2'—C6'—O1'	−179.38 (11)
C3—C2—C6—O1	−0.45 (17)	C3'—C2'—C6'—O1'	0.38 (17)
C1—C2—C6—C7	0.6 (2)	C1'—C2'—C6'—C7'	2.1 (2)
C3—C2—C6—C7	−179.61 (11)	C3'—C2'—C6'—C7'	−178.17 (12)
C9—C4—C7—C8	2.6 (2)	C9'—C4'—C7'—C8'	1.5 (2)
C5—C4—C7—C8	179.51 (11)	C5'—C4'—C7'—C8'	179.64 (11)
C9—C4—C7—C6	−177.01 (11)	C9'—C4'—C7'—C6'	−178.04 (11)
C5—C4—C7—C6	−0.12 (13)	C5'—C4'—C7'—C6'	0.10 (13)
O1—C6—C7—C4	1.95 (13)	O1'—C6'—C7'—C4'	0.57 (13)
C2—C6—C7—C4	−178.82 (12)	C2'—C6'—C7'—C4'	179.24 (12)
O1—C6—C7—C8	−177.67 (11)	O1'—C6'—C7'—C8'	−178.96 (11)
C2—C6—C7—C8	1.6 (2)	C2'—C6'—C7'—C8'	−0.3 (2)
O1—C5—C10—C15	90.54 (12)	O1'—C5'—C10'—C11'	−94.48 (12)
C4—C5—C10—C15	−21.21 (15)	C4'—C5'—C10'—C11'	154.11 (10)
C16—C5—C10—C15	−148.43 (10)	C16'—C5'—C10'—C11'	27.23 (14)
O1—C5—C10—C11	−83.48 (12)	O1'—C5'—C10'—C15'	81.20 (12)
C4—C5—C10—C11	164.77 (10)	C4'—C5'—C10'—C15'	−30.22 (14)
C16—C5—C10—C11	37.56 (14)	C16'—C5'—C10'—C15'	−157.09 (10)
C15—C10—C11—C12	0.68 (17)	C15'—C10'—C11'—C12'	1.69 (17)
C5—C10—C11—C12	174.74 (11)	C5'—C10'—C11'—C12'	177.34 (10)
C10—C11—C12—C13	−0.52 (18)	C10'—C11'—C12'—C13'	−1.61 (18)
C11—C12—C13—C14	−0.1 (2)	C11'—C12'—C13'—C14'	0.16 (19)
C12—C13—C14—C15	0.6 (2)	C12'—C13'—C14'—C15'	1.22 (19)
C13—C14—C15—C10	−0.41 (19)	C13'—C14'—C15'—C10'	−1.13 (19)
C11—C10—C15—C14	−0.22 (17)	C11'—C10'—C15'—C14'	−0.33 (17)
C5—C10—C15—C14	−174.24 (11)	C5'—C10'—C15'—C14'	−176.05 (11)
O1—C5—C16—C17	−30.62 (13)	O1'—C5'—C16'—C17'	−21.91 (15)
C4—C5—C16—C17	80.23 (12)	C4'—C5'—C16'—C17'	89.06 (12)
C10—C5—C16—C17	−149.77 (10)	C10'—C5'—C16'—C17'	−142.14 (11)
O1—C5—C16—C21	155.10 (10)	O1'—C5'—C16'—C21'	164.16 (10)
C4—C5—C16—C21	−94.05 (12)	C4'—C5'—C16'—C21'	−84.88 (13)
C10—C5—C16—C21	35.95 (15)	C10'—C5'—C16'—C21'	43.92 (15)
C21—C16—C17—C18	−1.44 (17)	C21'—C16'—C17'—C18'	0.08 (18)
C5—C16—C17—C18	−175.72 (10)	C5'—C16'—C17'—C18'	−173.88 (11)
C16—C17—C18—C19	−0.09 (19)	C16'—C17'—C18'—C19'	−0.94 (19)
C17—C18—C19—C20	0.7 (2)	C17'—C18'—C19'—C20'	0.8 (2)
C18—C19—C20—C21	0.3 (2)	C18'—C19'—C20'—C21'	0.1 (2)
C19—C20—C21—C16	−1.8 (2)	C19'—C20'—C21'—C16'	−1.0 (2)
C17—C16—C21—C20	2.41 (18)	C17'—C16'—C21'—C20'	0.86 (19)
C5—C16—C21—C20	176.74 (11)	C5'—C16'—C21'—C20'	174.90 (12)

### Hydrogen-bond geometry (Å, º)

Cg1 is the centroid of the C10–C15 phenyl ring

**Table:**

*D*—H···*A*	*D*—H	H···*A*	*D*···*A*	*D*—H···*A*
C20′—H20′···*Cg*1^i^	0.95	2.69	3.4041 (14)	133
C9—H9*B*···N3′	0.98	2.70	3.4560 (14)	134

Symmetry code: (i) −*x*+1, −*y*+1, −*z*.
